# Long-term outcomes of arthroscopic synovectomy and core decompression through multiple small bone holes for early-stage avascular necrosis of the femoral head

**DOI:** 10.1186/s42836-023-00181-8

**Published:** 2023-04-01

**Authors:** Quanbo Ji, Xiaoya Li, Song Luo, Lei Geng, Peng Ren, Ming Ni, Qingyuan Zheng, Peng Xin, Yan Wang, Guoqiang Zhang

**Affiliations:** Department of Orthopaedics, General Hospital of Chinese People’s Liberation Army, Beijing, 100853 China

**Keywords:** Synovectomy, Core decompression, Avascular necrosis of femoral head, Hip

## Abstract

**Objective:**

This study described a minimally invasive approach for the management of early-stage avascular necrosis of the femoral head, which integrated arthroscopic intra-articular decompression and core decompression by drilling multiple small holes.

**Method:**

A total of 126 patients with 185 hip avascular necrosis were included between March 2005 and January 2008, and the hips were classified, based on the Association Research Circulation Osseous staging system, into stage I (*n* = 43), stage II (*n* = 114), and stage III (*n* = 28). Arthroscopic intra-articular inspection and debridement, along with drilling of multiple small holes for core decompression, were performed. The Modified Harris hip score system and radiographs were used to assess the pre- and post-surgery outcomes.

**Results:**

One hundred and three patients (involving 153 hips) were followed up successfully for an average of 10.7 ± 3.4 years (range: 9–12 years). After surgery, the overall survival rate was 51.6% (79 hips), and the clinical survival rates were 79%, 72%, 52%, 32%, and 10% for patients with stage I, IIa, IIb, IIc, and III, respectively. The outcomes of patients with Association Research Circulation Osseous Stages I or IIA were better than those of other stages, while hips with a large necrotic area had poor results. This approach preserved the original biomechanical strength of the femoral head after core decompression and eliminated arthritis factors in the hip joint.

**Conclusion:**

The core decompression with multiple small-size holes is an effective method for treating early-stage avascular necrosis of the femoral head, particularly in those with pathological changes in the hip joint.

**Level of evidence:**

Therapeutic study, Level IV.

## Introduction

Avascular necrosis (AVN) of the femoral head has been one of the challenging research subjects [1–4]. Currently, core decompression of the hip represents the most common method for the treatment of early stage AVN [5]. However, what is the optimal decompression technique remains controversial.

The Association Research Circulation Osseous (ARCO) classification is a commonly used staging system for the assessment of AVN of the femoral head. The ARCO classifies ANV of the femoral head into stage 0 to VI. Among them, stage I to III refer to the early stages of AVN with normal joint space and virtually normal femoral head. There are many femoral head-preserving techniques, such as core decompression [6, 7], vascularized or non-vascularized bone grafting [8, 9], and osteotomy [10]. Smith et al*.* [11] reviewed 12 articles (702 hips) published between 1979 and 1991 on core decompression for the early stages of AVN. The successful rates of stage I, II, and III AVN were 78%, 62%, and 41%, respectively. However, the core decompression techniques vary widely with surgical approaches, the number of drill holes, and the diameter of the trephine. Many publications support multiple drilling as an appropriate core decompression alternative. Marker et al. [12] reviewed 1337 core decompression operations before 1992 and 1268 operations after 1992. They found the survival rates increased from 59% (range, 29–85%) to 70% (range, 39–100%) after a mean follow-up period of 38 months. However, the shortcomings of this conventional core decompression are the use of large-diameter trephine that weakens the supporting strength of the femoral head. It may overly damage the articular cartilage when the trephine is inserted into the joint space [13, 14]. In addition, synovitis and synovial fluid accumulation in the joint space may lead to further hip pain because the pathological changes are left untreated [15–17]. To improve the conventional techniques, we initially performed synovectomy, followed by core decompression through multiple small bone holes created on the femoral head. We hypothesized that this combined procedure might yield more favorable clinical outcomes.

This retrospective study aimed to use arthroscopic synovectomy and core decompression for the treatment of early-stage AVN of the femoral head. The decompression was performed through multiple small bone holes created on the femoral head. We also assessed the long-term outcomes of the treatment.

## Materials and methods

This project was a prospective single-arm case series study, and was approved by the institutional review boards of the hospitals involved. Informed consent and Health Insurance Portability and Accountability Act consent were obtained from all the patients.

Recruited were 168 patients with AVN of the femoral head who received treatment in the General Hospital of the People's Liberation Army (Beijing, China) from March 2005 to January 2008. Our inclusion criteria were (1) patients with early-stage AVN (ARCO stage I to III); (2) patients with either unilateral or bilateral involvement; (3) symptomatic patients who complained of pain, stiffness or limited range of motion in the affected joint, among others; (4) patients who had not undergone any previous surgical treatment for avascular necrosis; (5) patients who were not contraindicated for the proposed intervention or treatment being studied; (6) patients who provided informed consent to participate in the study; (7) patients who were willing and able to comply with the study protocol, including attending follow-up appointments and completing study-related assessments and (4) patients who failed to complete the entire follow-up. The study excluded patients with ARCO stage 0, as this stage typically involves asymptomatic AVN that tends to be treated non-surgically. For patients with ARCO stage I, diagnosis was based on findings of MRI and/or bone scintigraphy. Patients with ARCO stage II had radiographic evidence of sclerotic, cystic, or osteoporotic changes in the femoral head. Those with ARCO stage III showed a subchondral fracture on radiographs, often referred to as the "crescent sign". Patients with ARCO stage IV radiographically showed evidence of flattening of the femoral head, while those with ARCO stage V exhibited both flattening of the femoral head and osteoarthritic changes, such as decreased joint space and acetabular changes. Finally, patients with ARCO stage VI suffered from complete joint destruction. Patients with ARCO stage IV to VI were removed because a severe collapse of the femoral head could not be corrected/managed with minimally invasive treatments. Traumatic AVN was ruled out because assessment of the femoral head was probably difficult due to the anatomical changes. Finally, 126 eligible patients (185 hips) were treated with arthroscopy-assisted core decompression through multiple small bone tunnels. AVN of the femoral head was staged by a senior orthopedic surgeon. All operations were performed by the same surgical team.

### Surgical technique

**Fig. 1:**
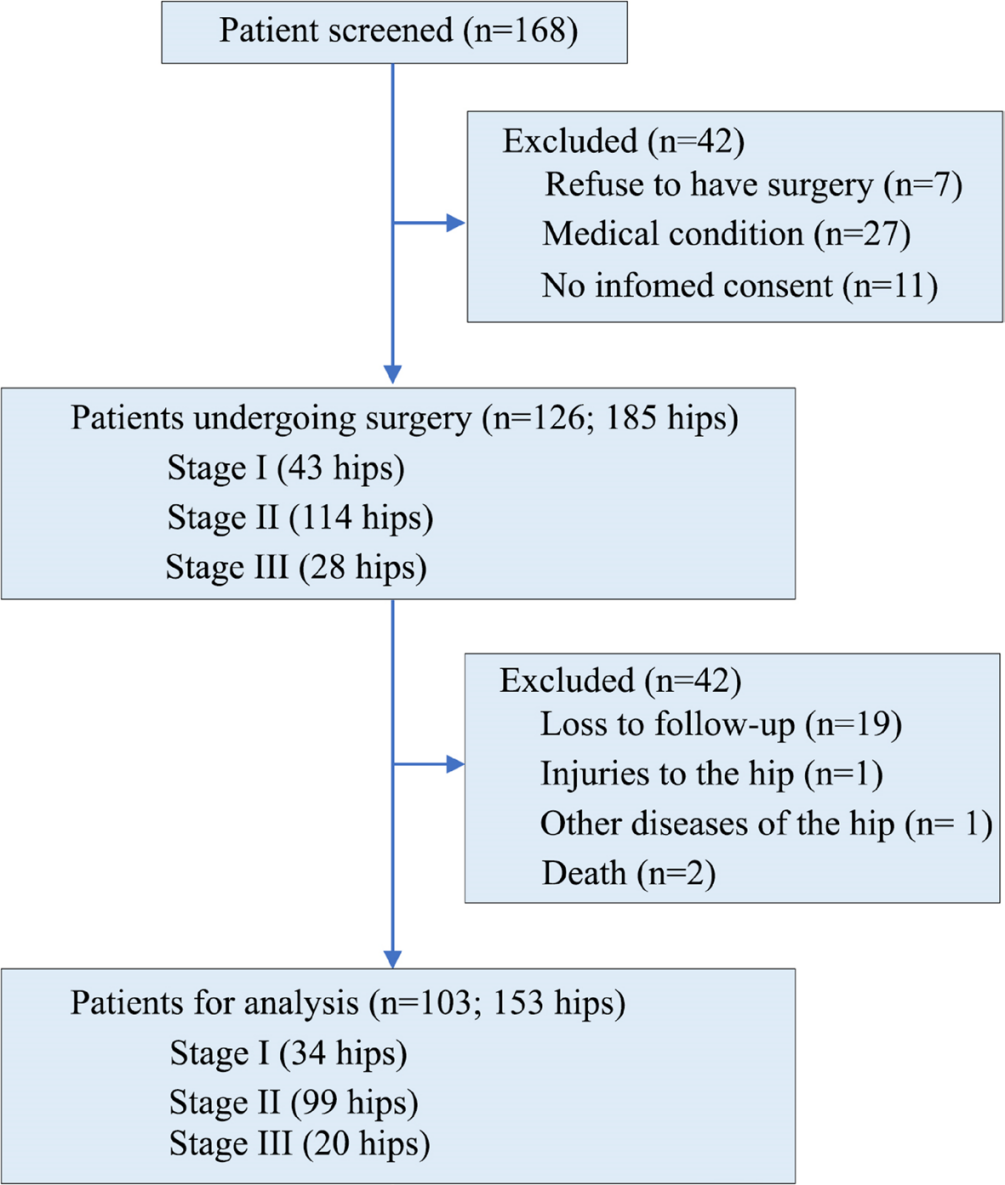
Arthroscopic photos showing hip synovitis. **A** Synovial hypertrophy; **B** Hyperemia; **C** Hyperplasia; **D** Synovectomy is complete

**Fig. 2:**
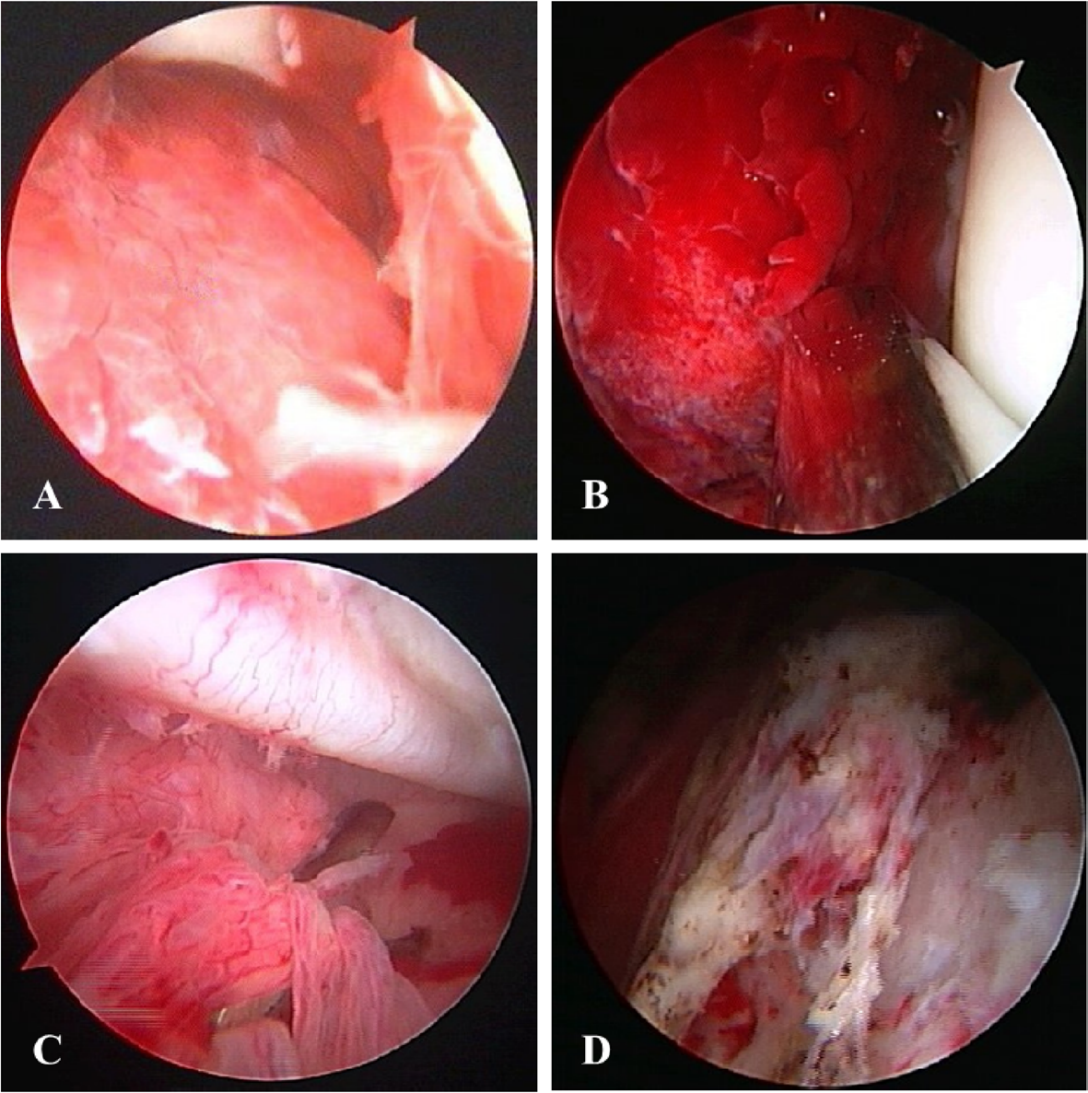
Arthroscopic photos of the right hip showing the cartilage lesions. **A** Acetabular cartilage damage; **B** Cartilage damage of femoral head

The operation was performed under general anesthesia. The supine position is preferred since it facilitates arthroscopy without entailing traction. We made three portals, *i*.*e*., an anterior peri-trochanteric portal, a posterior peri-trochanteric portal, and a lateral portal (2 cm proximal to the greater trochanter). Under fluoroscopic guidance, we introduced an 18 gauge, 25-cm long spinal needle from the lateral aspect of the hip into the joint, parallel to the femoral neck. We injected 10 mL of normal saline to expand the joint space and confirmed the needle position by checking continuous outflow. The needle facilitated the introduction of a 0.8-mm guide wire into the joint space, and the needle was then withdrawn. We introduced a cannulated obturator with a 5-mm arthroscopy cannula over the guide wire. Then, an anterolateral (or posterolateral) portal was created 2 cm anterior (or posterior) to the lateral portal in the same way. Other cannulae were introduced under fluoroscopic guidance. Then, a camera was introduced into the hip joint from the portal. The synovium in the superolateral, lateral, and inferolateral regions and surrounding the acetabular fossa was removed from another portal as needed (Fig. 1A, B, C, D). The synovial debris was removed with 8000 mL of normal saline. A drainage tube was placed through one of the portals. Cartilage lesions of the femoral head and acetabulum were assessed (Fig. 2A, B). The AVN area was located based on the preoperative X-ray. The articular cartilage over the AVN area was smoothed using the radio-frequency technique. During the procedure, the surgeon first made a small incision in the skin overlying the femoral head, and then inserted an arthroscope to visualize the interior of the joint. Using a 3-mm Steinmann pin, we made a drill hole in the AVN area. Under the fluoroscopic control to ensure proper placement, the surgeon then introduced a small drill or other instrument into the femoral head. The instrument was used to remove a small core of bone from the center of the femoral head, creating a channel for decompression. Under arthroscopic and fluoroscopic guidance, core decompression was performed either manually or by employing a low-speed power drill. This allowed for decompression of the necrotic bone and reduction of pressure within the femoral head. The procedure was performed arthroscopically to minimize tissue trauma and allow for faster recovery.

### Postoperative management

Rehabilitation exercises such as abductor strengthening and movement of the hip were encouraged two days after surgery. The patients were required to bear half of the body weight and walk with a cane or crutch held in the contralateral hand. The patients undergoing bilateral core decompression walked with two crutches. Six weeks after surgery, full weight-bearing was allowed as tolerated. However, high-impact loading movements such as jumping and landing were restricted. If the patients showed an asymptomatic hip and no radiographic evidence of head collapse 3 months after surgery, all usual activities, including high-impact loading activities, were allowed. The primary endpoint is typically the preservation of the femoral head and the prevention of the need for total hip replacement. Secondary endpoints include pain relief, improved range of motion, and functional status. The patient returned for follow-ups 3 months, 6 months, and every year after surgery. X-rays are useful for evaluating bone structure and detecting changes in bone density. MRI, on the other hand, can detect early changes in the bone and provide a more detailed view of the soft tissues surrounding the joint. Thus, X-rays or magnetic resonance imaging (MRI) were taken at the follow-up visits.

### Assessment

The patient's education level was rated, based on the International Standard Classification of Education, on a 0–8 scale, including low levels (0–3, early childhood education, primary education, and high school) and high levels (4–8, education beyond high school, including bachelor’s, master’s, and doctoral degree) [18]. The preoperative body mass index (BMI) was measured. The identification of the different stages of ARCO in the study is based on imaging scans. These imaging scans can provide detailed information about the extent and severity of avascular necrosis in the femoral head, which can be used for staging and grading against the ARCO classification system. Hip function was assessed in terms of the modified Harris Hip Score, with most limitations listed as 0 and no limitations as 100 [19]. Hip pain during weight-bearing was assessed on a 10-mm visual analogue scale (VAS) (0 mm, no pain; 10 cm, worst pain) [20]. Hip synovitis was diagnosed on the basis of the intraoperative findings, including synovial hyperplasia, hypertrophy, hyperemia, and swelling, and the severity was arthroscopically classified into grade I and II [21]. The extent of cartilage lesion of the femoral head and acetabulum was categorized into grade I and II based on arthroscopic evaluation [22]. Failed surgery was defined as the need for secondary surgery or a modified HHS less than 75 points. Recurrence of head collapse or increased collapse of more than 2 mm on X-ray was also deemed a failure. "Overall survival rate" refers to the percentage of patients who are still alive after a certain period of time, typically measured in years, following the diagnosis or treatment of a particular disease or condition. On the other hand, "clinical survival rates" refer to the percentage of patients who are still alive after a certain period of time, typically measured in years, following the diagnosis or treatment of a particular disease or condition.

### Statistical analysis

We compared the preoperative and postoperative Harris Hip Scores of the hip using a paired *t*-test. Survivorship analysis was performed by using the Kaplan–Meier method. A *P* < 0.05 (two-tailed) was considered statistically significant. The analyses were carried out using the SPSS version 22.0 (SPSS Inc, Chicago, IL, USA).

## Results

**Fig. 3:**
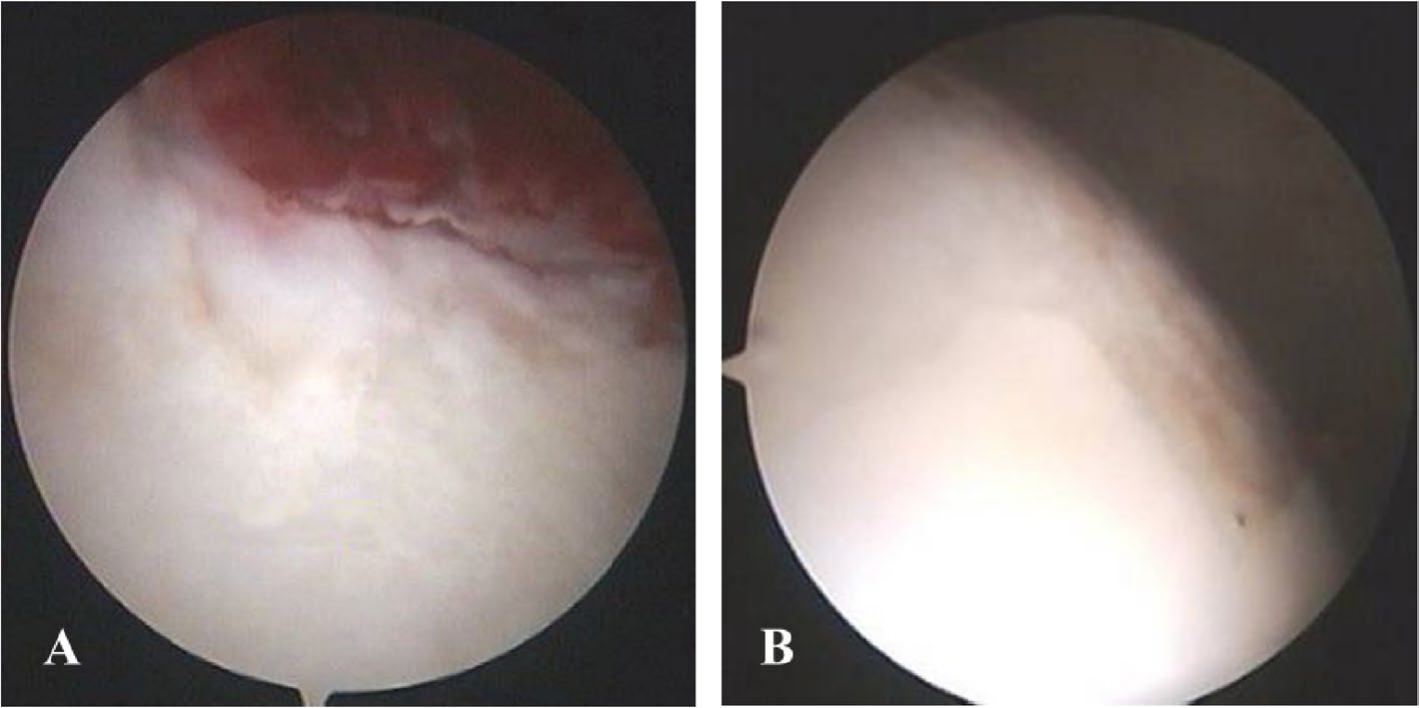
Flow diagram of 168 patients with avascular necrosis of the femoral head

Our study recruited 126 patients (185 hips involved) (Fig. 3), including 95 males and 31 females. The mean age at surgery was 38 years (range, 17–58 years) (Table 1). According to preoperative MRI, 43 hips were rated ARCO stage I, 114 stage II (114 hips), and 28 stage III. The mean preoperative modified HHS was 65.0 ± 10.1 and the mean VAS was 9.1 ± 0.5. Intraoperatively, hip synovitis was found in 153 (83%) hips, and cartilage lesion of the femoral head in 51 hips (Table 2). No wound infection, deep vein thrombosis, fracture developed and no revision decompression was performed.

**Table 1:** Demographics and preoperative characteristics of 126 patients (185 hips)

Age, mean (range; year)	38 (17–58)
Male: female (*n*)	95: 31
Right: left (*n*)	92: 93
Unilateral: bilateral involvement	89: 43
Education level, *n* (%)	
Low	55 (44)
High	71 (56)
Etiologic factors, *n* (hip)	
Alcohol abuse	53 (85)
Steroid use	48 (70)
Unknown causes	25 (30)
Body mass index	26 ± 6.2
< 25, *n* (%)	43 (34)
25–30, *n* (%)	65 (52)
> 30, *n* (%)	18 (14)
ARCO classification, hip (%)	
Stage I	43 (23)
Stage II	114 (62)
Stage III	28 (15)
Modified Harris Hip Score	65 ± 10.1
Visual analogue scale (mm)	9.1 ± 0.5

Data are shown as mean ± standard deviation

**Table 2:** Intraoperative findings in 126 patients (185 hips)

Synovitis: No Synovitis, Hip^a^	
Stage I	19: 24
Stage II	52: 31
Stage III	28: 0
Synovitis Severity, Hip (%)	
Grade I	86 (46)
Grade II	67 (36)
Cartilage Lesion of Femoral Head, Hip (%)	
Grade I	22 (11)
Grade II	29 (16)
Stage II^a^	23 (12)
Stage III^a^	28 (15)
Acetabular Cartilage Lesion, Hip (%)	51 (27)

^a^Intraoperative findings in Association Research Circulation Osseous (ARCO) stage I to III

In our series, 23 patients (32 hips; including 9 ARCO stage I, 15 stage II, and 8 stage III AVN) lost to follow-up. A total of 103 patients (153 hips) completed the follow-ups, and the mean follow-up period lasted 10.7 ± 3.4 years (range, 9–12 years).At the final follow-up, the overall survival rate was 52% (79 hips) (Table 3). The clinical survival rates of stage I, IIa, IIb, IIc, and III AVN were 79%, 72%, 52%, 32%, and 10%, respectively (Table 4). All 20 hips with ARCO stage III AVN were completely followed up, in which 2 hips scored 80 points on modified HHS scale and the daily activities were not affected. Surgery failed in 74 hips, including 7, 49 and 18 hips with ARCO stage I, II and III AVN, respectively. The mean modified HHS was 70.15 ± 4.3, and the mean VAS was 9.1 ± 0.5. There were significant differences between preoperative and postoperative modified HHS (*P* = 0.02) and VAS (*P* = 0.01).

**Table 3:** Outcomes of 103 patients (153 hips) at the final follow-up

Follow-up, mean (range), year	10.7 ± 3.4 (9–12)
Modified Harris Hip Score	70.15 ± 4.3
Visual analogue scale (mm)	9.1 ± 0.5
Survival rate, hip (%)	79 (51)

Data are shown as mean ± standard deviation

**Table 4:** Clinical and radiographic outcomes categorized by the Association Research Circulation Osseous (ARCO) stage of avascular necrosis

Stage	I	IIa	IIb	IIc	III
Hip undergoing surgery (*n*)	43	33	41	40	28
Hip at the last follow up (*n*)	34	28	35	36	20
Clinical survival (*n*)	27	20	18	12	2
Clinical failure					
Modified Harris Hip Score < 75 (*n*)	2	4	7	8	9
Secondary surgery (*n*)	5	4	10	16	9
Clinical survival rate (%)	79	72	52	32	10
Radiographic survival (*n*)	24	20	16	8	2
Radiographic failure					
Additional collapse (*n*)	5	4	9	12	9
Secondary surgery (*n*)	5	4	10	16	9
Radiographic survival rate (%)	71	72	45	23	10

The modified HHSs categorized by the stages of AVN are shown in Table 5. Synovial fluid accumulation improved significantly on MRI. The necrotic area of the femoral head was minimized, and the edema alleviated.

**Table 5:** Comparison of modified Harris Hip Scores (categorized by the ARCO) measured preoperatively and at the last follow-up

ARCO	Time	Modified HHS	*t*	*P* Value
Stage I	Before operation	70.15 ± 4.3	-18.88	< 0.01
	Last follow-up	93.33 ± 5.92		
Stage II	Before operation	68.5 ± 8.3	-14.73	< 0.01
	Last follow-up	87.3 ± 5.5		
Stage III	Before operation	56.7 ± 11.2	-7.03	< 0.01
	Last follow-up	84.4 ± 7.8		

*ARCO* Association Research Circulation Osseous, *HHS* Harris Hip Score; Data are shown as mean ± standard deviation

Treatment failures due to aggressive collapse (> 2 mm) happened in 30 patients (44 hips). Among them, most treatments (34 hips) failed within 5 years, and the failure rate decreased rapidly thereafter. Secondary total hip replacement was performed in 29 hips, and vascularized bone grafting in 5 hips. The remaining 7 patients (10 hips) declined to receive a second surgery due to the cost-related concern.

## Discussion

In this study, the intra-articular decompression through hip arthroscope plus core decompression by drilling multiple small holes was found to be an effective method for treating early-stage avascular necrosis of the femoral head, particularly in those with pathological changes in the hip joint.

AVN of the femoral head is a condition where the blood supply to the femoral head is reduced. The condition might result from traumatic or non-traumatic factors and eventually leads to the collapse of the femoral head [1, 23, 24]. Furthermore, AVN of the femoral head is linked to pathological synovial reaction, hypertrophy, effusion, and an increase in intra-articular pressure [2, 4, 25–28]. To understand the correlation between intra-articular pressure of the hip joint and blood flow to the femoral head in animal models, a microsphere of radioactively-labeled red cells was used. Additionally, the extent of blood supply to the femoral head was quantitatively assessed using the hydrogen washout technique. The results revealed a positive correlation between the increased intra-articular and intra-osseous pressure, which led to a reduced blood flow to the femoral head. The data provided evidence that lowering the intra-articular and intra-osseous pressure might be a treatment option for AVN.

The ARCO system is based on the extent of AVN in the femoral head, as determined by imaging studies such as MRI or CT scans. The system assigns a stage to a patient based on the size of the necrotic lesion, the location of the lesion within the femoral head, and the presence or absence of subchondral collapse. The system also contains a grading system to rate the severity of the disease on the basis the degree of subchondral collapse and the extent of femoral head involvement. In this study, we chose the ARCO system for several reasons. First, the ARCO system is widely recognized and used by clinicians and researchers to classify and stage AVN, which renders it easier to compare results across studies. Second, the ARCO system provides a standardized method for evaluating the extent and severity of AVN, which informs treatment decisions and outcome prediction. Finally, the ARCO system is relatively easy to use and applicable to most imaging studies, which makes it a practical tool for clinical research.

While hip arthroscopy was first introduced in 1931, its extensive application has been hampered by the difficult accessibility of the hip joint. With advances in surgical technique and instruments, the procedure has become increasingly doable and feasible for surgeons. As a result, hip arthroscopy has gained popularity in recent years, consistently accomplishing better clinical outcomes [14, 16, 29, 30]. Our clinical case series demonstrated that hip arthroscopy allows clinicians to comprehensively evaluate the hip joint, thereby facilitating the removal of all inflamed synovial tissues by washing. This approach especially benefited patients who exhibited noticeable synovitis in the hip joint. Our study yielded better clinical outcomes when compared to previous reports.

Opening channels for re-vascularization of the femoral head via core decompression has been seen as a working strategy for decreasing intra-osseous pressure in the initial stage of AVN [6, 31]. Arthroscopic intra-articular decompression prevents femoral head collapse by removing loose bodies and debris from the joint space. This procedure involves the use of a small camera to visualize the hip joint's interior and remove any damaged or diseased tissue, which can mitigate inflammation and promote healing. Additionally, removing debris can enlarge the joint space, thus reducing pressure on the femoral head. Due to the femoral head's spherical shape, the extent of core decompression under arthroscopic control cannot be accurately assessed without intra-articular monitoring. Lin et al. conducted a study using a fresh adult cadaveric femur specimen to investigate the impact of drilling holes of different diameters on the structural properties of femoral head. They found that larger pore diameter and single tract core decompression could significantly weaken the femoral head. On the other hand, the use of multiple small diameter (2.5–3.0 mm) low-speed drillings could equally achieve core decompression and opening of vascular channels while preserving the biomechanical strength of the femoral head. However, the precise placement of the drillings without penetrating the cortex could only be accomplished under arthroscopic guidance. Ruch et al. reported favorable outcomes using hip arthroscopy to guide the placement of core decompression in the hip [32]. In this study, our result suggested that the arthroscopic intra-articular decompression in combination with core decompression using multiple small drill holes could effectively reduce pressure on the femoral head, thus preserving its structural integrity and obviating collapse.

Our study has several limitations that need to be addressed. Firstly, we didn't compare arthroscopic intra-articular decompression and drilling decompression using conventional surgical techniques such as Kirschner wire for avascular necrosis of femoral head. Further research is warranted to make such comparison. Secondly, due to the large number of patients, the surgeries were performed by the same surgical team but not by the same surgeon. Hence, the effect of the techniques might be influenced by surgeons' preference, experience, and operative skills. Additionally, a long-term follow-up and a larger sample size are needed to identify the functions and complications associated with this surgical technique. 

## Conclusion

The combination of arthroscopic synovectomy and core decompression through multiple small bone holes is a safe and efficacious surgical technique for the treatment of early stages of AVN of femoral head, and might achieve favorable long-term outcomes.

## Data Availability

All data and materials are available on reasonable request.
